# MIND, BODY, AND SOUL: PURPOSE IN LIFE AS A MEDIATOR BETWEEN PHYSICAL ACTIVITY AND COGNITIVE AGING

**DOI:** 10.1093/geroni/igad104.3470

**Published:** 2023-12-21

**Authors:** Farima Sadeqi, Ryan Best

**Affiliations:** West Virginia University, Morgantown, West Virginia, United States; West Virginia University, Morgantown, West Virginia, United States

## Abstract

Recent guidelines from the WHO stress the benefits of physical activity and harm of sedentary behavior across the lifespan. Physical activity is associated with multiple positive life outcomes, both physical and psychological, across adulthood and into old age. Previous studies have shown engaging in aerobic physical activity buffers cognitive decline and can even enhance cognition in older adulthood. Compared to the growing literature on physiological links between physical activity and cognitive aging, there is a gap in the literature investigating psychological pathways. Empirical research suggests a positive bidirectional association between purpose in life and engagement in physical activity across adulthood. Furthermore, recent research has provided some evidence that purpose in life, both subjectively and objectively, is a positive predictor of cognitive functioning. Merging these areas of research, we investigated purpose in life as a mediator in the relationship between physical activity and cognitive aging. Using the MIDUS study, a representative sample of adults aged 25 to 74 years, a mediation model was fit to the data to test a psychological mechanism explaining the relationship between physical activity and cognition in adults. Physical activity was categorized into three groups based on the intensity of the activity—light, moderate, and vigorous. A small, significant mediation was present across all levels of activity with light physical activity showing a slightly stronger indirect effect when compared to the other two. Results will be discussed considering levels of physical activity and the implication of differential psychological and physiological pathways describing downstream effects on cognitive aging.

